# Upregulation of cellular glutathione levels in human *ABCB5*- and murine *Abcb5*-transfected cells

**DOI:** 10.1186/s40360-015-0038-5

**Published:** 2015-12-15

**Authors:** Shingo Kondo, Keita Hongama, Kengo Hanaya, Ryota Yoshida, Takaaki Kawanobe, Kazuhiro Katayama, Kohji Noguchi, Yoshikazu Sugimoto

**Affiliations:** Division of Chemotherapy, Faculty of Pharmacy, Keio University, 1-5-30 shibakoen, Minato-ku, 105-8512 Tokyo Japan; Division of Organic and Biocatalytic Chemistry, Faculty of Pharmacy, Keio University, 1-5-30 shibakoen, Minato-ku, 105-8512 Tokyo Japan

**Keywords:** ABC transporter, ABCB5, ABCB1, P-glycoprotein, Drug resistance, Buthionine sulfoximine, Cancer stem cell, Glutathione synthesis

## Abstract

**Background:**

Previously, we have demonstrated that human ABCB5 is a full-sized ATP-binding cassette transporter that shares strong homology with ABCB1/P-glycoprotein. *ABCB5*-transfected cells showed resistance to taxanes and anthracyclines. Herein, we further screened ABCB5 substrates, and explored the mechanism of resistance.

**Methods:**

Sensitivity of the cells to test compounds was evaluated using cell growth inhibition assay. Cellular levels of buthionine sulfoximine (BSO), glutathione and amino acids were measured using HPLC and an enzyme-based assay. Cellular and vesicular transport of glutathione was evaluated by a radiolabeled substrate. Expression levels of glutathione-metabolizing enzymes were assessed by RT-PCR.

**Results:**

Human *ABCB5*-transfected 293/B5-11 cells and murine *Abcb5*-transfected 293/mb5-8 cells showed 6.5- and 14-fold higher resistance to BSO than the mock-transfected 293/mock cells, respectively. BSO is an inhibitor of gamma-glutamylcysteine ligase (GCL), which is a key enzyme of glutathione synthesis. 293/B5-11 and 293/mb5-8 cells also showed resistance to methionine sulfoximine, another GCL inhibitor. A cellular uptake experiment revealed that BSO accumulation in 293/B5-11 and 293/mb5-8 cells was similar to that in 293/mock cells, suggesting that BSO is not an ABCB5 substrate. The cellular glutathione content in 293/B5-11 and 293/mb5-8 cells was significantly higher than that in 293/mock cells. Evaluation of the BSO effect on the cellular glutathione content showed that compared with 293/mock cells the BSO concentration required for a 50 % reduction in glutathione content in 293/B5-11 and 293/mb5-8 cells was approximately 2- to 3-fold higher. This result suggests that the BSO resistance of the *ABCB5*- and *Abcb5*-transfected cells can be attributed to the reduced effect of BSO on the transfectants. Cellular and vesicular transport assays showed that the transport of radiolabeled glutathione in 293/B5-11 cells was similar to that in 293/mock cells. The mRNA expression of genes encoding glutathione-metabolizing enzymes in 293/B5-11 cells was similar to that in 293/mock cells. The cellular content of Glu, a precursor of glutathione, in 293/B5-11 and 293/mb5-8 cells was higher than that in 293/mock cells.

**Conclusions:**

*ABCB5*/*Abcb5*-transfected cells showed resistance to BSO, which is not a substrate of ABCB5. Our results suggest that ABCB5/Abcb5 upregulates cellular glutathione levels to protect cells from various poisons.

## Background

Human ATP-binding cassette (ABC) transporters comprise a superfamily of 48 members, which are classified into seven subfamilies (ABCA–ABCG) according to sequence homology. ABCB1/P-glycoprotein is expressed as a 170–180 kDa transmembrane glycoprotein that consists of two homologous halves, each containing a hydrophobic region with six transmembrane segments and a nucleotide-binding region [1–3]. ABCB1 functions as an efflux pump for various structurally unrelated anticancer drugs such as *vinca* alkaloids, anthracyclines and taxanes [1, 2, 4]. ABCB1 is expressed in a variety of normal tissues and cells, and plays a key role in the excretion of various natural compounds and xenobiotics [2]. Expression of ABCB1 in the apical surface of capillary endothelial cells in the brain and testis suggests a protective role of this transporter against toxic substances [2]. ABCG2/BCRP forms a homodimer bridged by a disulfide bond and mediates resistance to 7-ethyl-10-hydroxycamptothecin, topotecan and mitoxantrone [5, 6]. ABCG2 is expressed in hematopoietic stem cells and is a determinant of the side-population phenotype [7]. ABCC1/MRP1 mediates resistance to anthracyclines, *vinca* alkaloids, folate-based antimetabolites and etoposide [8]. ABCC1 is expressed in the gastrointestinal tract, liver, kidney and capillary endothelial cells, and mediates the efflux of a variety of organic anions, including glutathione and glucuronide conjugates, as well as unconjugated organic anions such as reduced glutathione (GSH) and folate derivatives. These findings suggest that ABC transporters have two major roles: (1) transporting natural substances and xenobiotics across the lipid bilayer membrane, and (2) protecting important organs and cells such as the brain, testis, and hematopoietic and tissue stem cells from toxic substances.

Previously, we have reported that human ABCB5 is a full-sized ABC transporter that consists of two homologous halves, each including a hydrophobic region with six predicted transmembrane segments and a nucleotide-binding region, and that it shares strong homology with ABCB1. ABCB5 confers resistance to taxanes and anthracyclines [9]. The cellular uptake of radiolabeled paclitaxel and docetaxel by the transfectants was lower than that by the parental cells. Membrane vesicles prepared from ABCB5 baculovirus-infected Sf21 cells showed high vanadate-sensitive ATPase activity that was sensitive to docetaxel [9]. Expression of full-length ABCB5 has been observed in the prostate and testis. In addition, it has been reported that ABCB5 is expressed in human melanoma tumor-initiating cells [10]. ABCB5-positive melanoma cells inoculated into immunodeficient mice showed greater tumorigenic capacity than ABCB5-negative cells [10]. Recently, murine Abcb5 expression has been reported in limbal stem cells, and was required for corneal development and repair [11]. These results suggest that ABCB5 may also have a protective function in stem cells.

In this study, human *ABCB5*- and murine *Abcb5*-transfected cells showed resistance to buthionine sulfoximine (BSO), an inhibitor of gamma-glutamylcysteine ligase (GCL), which is required for the first step of glutathione synthesis. A transport study showed that BSO is not a substrate of ABCB5. Our results showed that ABCB5/Abcb5 upregulates cellular glutathione levels. The BSO resistance of 293/B5-11 and 293/mb5-8 cells can be attributed to the lower effect of BSO on the depletion of cellular glutathione content.

## Methods

### Plasmids

Murine *Abcb5* cDNA (GenBank ID: NM_029961) was isolated by PCR using mouse testis cDNA (Takara, Ohtsu, Japan) as a template. The 5'-fragment of *Abcb5* cDNA was amplified using the primers, -79F (5'-GGAGAAAAGCCACACACGAA-3') and 1853R (5'-TAGTACAGCCCCTGCTTTGC-3'). The 3'-fragment of *Abcb5* cDNA was amplified using the primers, 1570F (5'-GCTCAAATGAGTGGAGGCCA-3') and 3791R (5'-CAGTGCACCCAATGAAGCAAT-3'). A c-Myc epitope tag was added to the N-terminus of the coding region by PCR. The two *Abcb5* cDNA fragments were sequenced, digested with XhoI, ligated and cloned into the bicistronic expression plasmid, pCAL-IRES-ZEO [12]. The resulting plasmid was termed pCAL-MycAbcb5-IRES-ZEO.

### Cells, transfectants and cell growth inhibition assay

Cells were cultured in Dulbecco’s modified Eagle’s medium supplemented with 7 % fetal bovine serum at 37 °C in 5 % CO_2_. Establishment of human *ABCB5*-transfected cells has been described previously [9]. To generate murine *Abcb5*-transfected cells, HEK293 cells were transfected with pCAL-MycAbcb5-IRES-ZEO using the FuGENE HD transfection reagent (Promega, Madison, WI, USA), and then selected with 50 μg/mL of zeocin for 8 days. Clonal cells were obtained from the mixed population by a standard limiting dilution technique. The sensitivity of the transfectants to anticancer agents was evaluated using a cell growth inhibition assay. Cell numbers were determined using a Coulter counter (Beckman Coulter, Brea, CA, USA).

### Western blot analysis

Protein expression was evaluated by western blotting as previously described [13]. Expression of Myc-tagged human ABCB5 and murine Abcb5 was evaluated using mouse anti-c-Myc monoclonal antibody 9E10 (Roche Diagnostics, Indianapolis, IN, USA). Mouse anti-glyceraldehyde 3-phosphate dehydrogenase (GAPDH) monoclonal antibody (Chemicon, Temecula, CA, USA) was used as a protein loading control. The blots were incubated with peroxidase-conjugated secondary antibodies (GE Healthcare, Little Chalfont, UK). The membrane-bound antibodies were visualized using the SuperSignal West Dura Extended Duration Substrate (Thermo Fisher Scientific, Waltham, MA, USA).

### Transport assay

Intracellular accumulation of BSO was quantified using an HPLC. Cells were plated at 5 × 10^6^ cells in a 100-mm dish and incubated at 37 °C for 18 h. After attachment to the plates, cells were incubated with 500 μM BSO for 0, 1 and 3 h. The untreated and BSO-treated cells were harvested, washed and lysed by addition of 1 mL of ethanol. The cell debris was removed by centrifugation at 18,000 × *g* for 20 min. The amine-containing compounds including BSO in the cell extracts were reacted with the fluorescent derivatizing reagent, 6-aminoquinolyl-N-hydroxysuccinimidyl carbamate (AQC; Waters, Milford, MA, USA) [14]. The resulting fluorescent derivatives were separated by HPLC on a 4.6 × 250 mm ID Inertsil ODS3 column (GL Sciences, Tokyo, Japan). Mobile phase A consisted of 50 mM sodium acetate and 1 % tetrahydrofuran, pH 6.6. Mobile phase B was methanol. The samples were applied onto the column and eluted at 65 °C at a flow rate of 1 mL/min by the following gradient: 0–25 min, 15–80 % B; 25–26 min, 80–100 % B; 26–46 min, 100 % B. The fluorescent compounds were detected using a Shimadzu RF-10A fluorescence detector (Shimadzu, Kyoto, Japan) with 250 nm excitation and 395 nm emission.

Transport of GSH was evaluated by cellular and vesicular transport assays using [2-glycine-^3^H]GSH (49.5 Ci/mmol; American Radiolabeled Chemicals, St. Louis, MO, USA). For the cellular uptake experiment, the cells (10^6^/tube) were incubated with 1 nM [^3^H]GSH at 37 °C for 0, 2, 5 and 10 min in Hanks’ balanced salt solution. The reaction was terminated by addition of ice-cold phosphate-buffered saline. After washing, the radioactivity in the cells was determined by a liquid scintillation counter. For the vesicular transport experiment, membrane vesicles were prepared according to a method described previously [13]. The vesicles (25 μg/tube) were incubated with 66 nM [^3^H]GSH in the absence or presence of 3 mM ATP at 25 °C for 0, 2 and 10 min in a reaction mixture containing 250 mM sucrose, 10 mM HEPES, 10 mM MgCl_2_, 10 mM phosphocreatine and 100 μg/mL creatine kinase. The reaction was terminated by addition of ice-cold stop solution (250 mM sucrose, 10 mM HEPES and 100 mM NaCl) and centrifuged at 18,000 × *g* for 10 min. After washing, the radioactivity in the membrane vesicles was determined by a liquid scintillation counter.

### Determination of cellular glutathione content

Cellular GSH content was measured using an HPLC. Cells were harvested and lysed by addition of methanol. The cell debris was removed by centrifugation at 18,000 × *g* for 20 min. The supernatant was derivatized using AQC and quantified by an HPLC. The HPLC column, flow rate, temperature, mobile phase A and B were the same as in the BSO uptake experiment. The gradient system was as follows: 0–75 min, 5–35 % B; 75–76 min, 35–100 % B; 76–101 min, 100 % B. The fluorescent compounds were detected using a Shimadzu RF-10A fluorescence detector with 250 nm excitation and 395 nm emission.

The effect of BSO on the cellular glutathione content was measured using a glutathione assay kit (Cayman Chemical, Ann Arbor, MI, USA) according to the manufacturer’s protocol [15]. Briefly, cells were incubated with various concentrations of BSO for 2 days. The cell extract was prepared, deproteinized and reacted with 5,5'-dithiobis(2-nitrobenzoic acid). In this reaction, glutathione reductase was added to convert glutathione disulfide to GSH. The resulting 5-mercapto-2-nitrobenzoic acid was quantified colorimetrically.

### mRNA expression analysis

Total RNA was extracted from cells using an RNeasy kit (Qiagen, Valencia, CA, USA) and subjected to a cDNA microarray analysis using SurePrint G3 Human GE 8 × 60 K Microarray (Agilent Technologies, Santa Clara, CA, USA). The mRNA expression of genes encoding enzymes involved in glutathione metabolism was also evaluated by RT-PCR. The genes analyzed in this study were *glutamate-cysteine ligase catalytic subunit* (*GCLC*), *glutamate-cysteine ligase modifier subunit* (*GCLM*), *glutathione synthase* (*GSS*), *glutathione reductase* (*GSR*) and *glutathione S-transferase pi 1* (*GSTP1*). Primer sequences are listed in Table 1. RT-PCR reactions were performed using an RNA LA PCR kit (Takara). The PCR conditions were as follows: 94 °C for 5 min, then increasing cycle numbers of 94 °C for 30 s, 51 °C for 30 s and 72 °C for 1 min, and a final extension step at 72 °C for 7 min.

**Table 1:** Sequences of primers used in RT-PCR analysis

Gene	Primer orientation	Sequence
GCLC	forward	5'-GTTCTCAAGTGGGGCGATGA-3'
	reverse	5'-CCGGCTTAGAAGCCCTTGAA-3'
GCLM	forward	5'-AAGTGCAGTTGACATGGCCT-3'
	reverse	5'-TGACCGAATACCGCAGTAGC-3'
GSS	forward	5'-GACCAGCGTGCCATAGAGAA-3'
	reverse	5'-ACCCTCTCTCTGGGGCTTTA-3'
GSR	forward	5'-GTGGCACTTGCGTGAATGTT-3'
	reverse	5'-AGTTTTCGGCCAGCAGCTAT-3'
GSTP1	forward	5'-CAAGTTCCAGGACGGAGACC-3'
	reverse	5'-GCCATTGATGGGGAGGTTCA-3'
GAPDH	forward	5'-ATCACCATCTTCCAGGAGCGA-3'
	reverse	5'-GCTTCACCACCTTCTTGATGT-3'

### Determination of cellular amino acids’ content

Cells were incubated at 37 °C for 4 h in fresh medium, harvested and lysed by addition of methanol. The cell debris was removed by centrifugation at 18,000 × *g* for 20 min. The supernatant was derivatized using 4-fluoro-7-nitro-2,1,3-benzoxadiazole (Dojindo, Kumamoto, Japan) [16]. The resulting fluorescent derivatives were separated by an HPLC on a 1.5 × 150 mm CAPCELL PAK C18 MGII S5 column (Shiseido, Tokyo, Japan). Mobile phase A consisted of 10 mM citric acid and 75 mM sodium perchlorate, pH 6.2. Mobile phase B was 50 % acetonitrile. The samples were applied onto the column and eluted at 50 °C at a flow rate of 0.5 mL/min with the following gradient: 0–20 min, 3–10 % B; 20–45 min, 10–37 % B; 45–50 min, 37–38 % B; 50–60 min, 38–79 % B; 60–65 min, 79–90 % B; 65–66 min, 90–100 % B; 66–70 min, 100 % B. The fluorescent compounds were detected using a Shimadzu RF-10A fluorescence detector with 480 nm excitation and 530 nm emission.

## Results

### Resistance of *ABCB5*- and *Abcb5*-transfected cells to GCL inhibitors

**Fig. 1:**
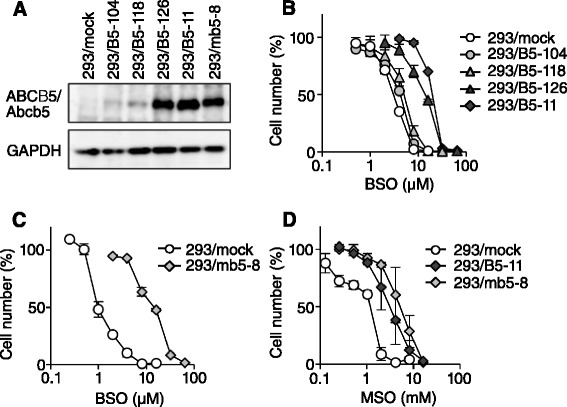
Resistance of *ABCB5*- and *Abcb5*-transfected cells to BSO and MSO. **a** Immunoblot analysis of the expression of Myc-tagged human ABCB5 and murine Abcb5 in the transfectants using anti-c-Myc monoclonal antibody 9E10. **b** Sensitivity of human *ABCB5*-transfected cells to BSO. Cells were cultured for 5 days in the absence or presence of various concentrations of BSO. Cell numbers were determined using a Coulter counter. The data are presented as mean ± SD of triplicate determinations. **c** Sensitivity of murine *Abcb5*-transfected cells to BSO. **d** Sensitivity of *ABCB5*- and *Abcb5*-transfected cells to MSO

We have previously reported that human *ABCB5*-transfected cells showed resistance to anthracyclines and taxanes [9]. In the present study, we tested the sensitivity of the parental and human *ABCB5*-transfected cells to various anticancer agents and cytotoxic compounds, and found that the *ABCB5*-transfected cells showed more resistance to BSO than the control cells. BSO is an inhibitor of GCL, a rate-limiting enzyme of glutathione synthesis. 293/B5-11 and 293/B5-126 cells, which expressed high amounts of ABCB5, showed 6.5- and 4.6-fold higher resistance to BSO than 293/mock cells, respectively, whereas 293/B5-104 and 293/B5-118 cells, which expressed low amounts of ABCB5, showed only 1.4- and 1.7-fold higher resistance to BSO than 293/mock cells, respectively (Fig. 1a and b). To confirm that the BSO resistance of *ABCB5* transfectants was due to the transporter function, we generated murine *Abcb5*-transfected HEK293 cells. The expression of Abcb5 in the zeocin-resistant clones was examined by western blotting using an anti-c-Myc antibody, and clone 293/mb5-8, which showed the highest Abcb5 expression was selected for further experiments (Fig. 1a). The molecular weight of human ABCB5 and murine Abcb5 is approximately 140 kDa, though the molecular weight of murine Abcb5 is slightly higher than that of human ABCB5. Compared with the control, 293/mb5-8 cells showed 14-fold higher resistance to BSO (Fig. 1c). We next examined the sensitivity of the *ABCB5*- and *Abcb5*-transfected cells to methionine sulfoximine (MSO), another GCL inhibitor with a similar structure to BSO. As shown in Fig. 1d, 293/B5-11 and 293/mb5-8 cells showed 3.0- and 4.5-fold higher resistance to MSO than 293/mock cells, respectively. These results indicate that the expression of ABCB5/Abcb5 confers resistance to GCL inhibitors.

### Intracellular accumulation of BSO in *ABCB5*- and *Abcb5*-transfected cells

**Fig. 2:**
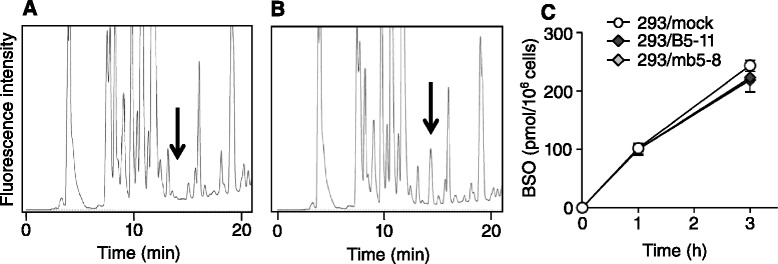
Intracellular accumulation of BSO in *ABCB5*- and *Abcb5*-transfected cells. **a** HPLC chromatogram of AQC-derivatized ethanol extract of 293/mock cells without incubation with BSO. **b** HPLC chromatogram of AQC-derivatized ethanol extract of 293/mock cells after a 3-h incubation with 500 μM BSO. A BSO peak indicated by an arrow was detected at 14.3 min. **c** Intracellular accumulation of BSO. 293/mock, 293/B5-11 and 293/mb5-8 cells were incubated with 500 μM BSO for 1 and 3 h. The intracellular BSO content was determined by HPLC. The data are presented as mean ± SD of triplicate determinations

To explore the mechanism of BSO resistance, we measured BSO accumulation in the *ABCB5*- and *Abcb5*-transfected cells. 293/mock, 293/B5-11 and 293/mb5-8 cells were incubated with 500 μM BSO for 1 and 3 h, and cell extracts were derivatized using AQC and subjected to HPLC analysis. The HPLC chromatograms of the 293/mock cells before the addition of BSO and 3 h after the addition of BSO are shown in Fig. 2a and b, respectively. A BSO peak indicated by an arrow was detected at 14.3 min (Fig. 2b), whereas no peak was detected around 14–15 min in the chromatogram without BSO treatment (Fig. 2a). As shown in Fig. 2c, BSO accumulation in 293/B5-11 and 293/mb5-8 cells was similar to that in 293/mock cells. This demonstrated that the BSO resistance of 293/B5-11 and 293/mb5-8 cells was not caused by reduced accumulation of BSO.

### Cellular glutathione in *ABCB5*- and *Abcb5*-transfected cells

**Fig. 3:**
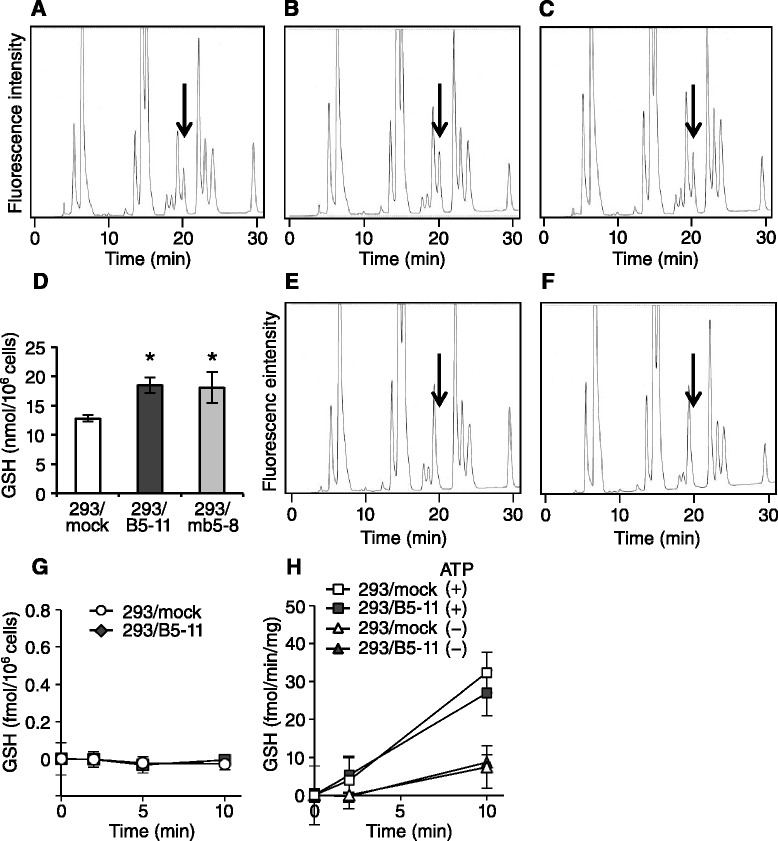
Cellular GSH content of *ABCB5*- and *Abcb5*-transfected cells. **a**–**c** HPLC chromatograms of AQC-derivatized methanol extracts of 293/mock (**a**), 293/B5-11 (**b**) and 293/mb5-8 (**c**) cells. GSH peaks indicated by arrows were detected at 20 min. **d** Cellular GSH content in 293/mock, 293/B5-11 and 293/mb5-8 cells determined by HPLC. The data are presented as mean ± SD of triplicate determinations. Asterisks show *p* < 0.05 by two-sided *t*-test. **e**, **f** HPLC chromatograms of AQC-derivatized methanol extracts of 293/mock (**e**) and 293/B5-11 (**f**) cells after incubation for 2 days in the presence of 100 μM BSO. **g** Intracellular accumulation of GSH in 293/mock and 293/B5-11 cells. Cells were incubated with 1 nM [^3^H]GSH at 37 °C for 0, 2, 5 and 10 min. **h** ATP-dependent uptake of GSH in 293/mock and 293/B5-11 vesicles. The membrane vesicles were incubated at 25 °C with 66 nM [^3^H]GSH in the absence or presence of 3 mM ATP for 0, 2 and 10 min

We next measured the cellular GSH content in *ABCB5*- and *Abcb5*-transfected cells using HPLC after derivatization with AQC. A GSH peak was detected at 20 min in the chromatogram of 293/mock cells (Fig. 3a, peak under arrow). The GSH peaks in the chromatograms of 293/B5-11 (Fig. 3b) and 293/mb5-8 (Fig. 3c) cells were higher than those of 293/mock cells. The cellular GSH content in 293/mock, 293/B5-11 and 293/mb5-8 cells was 12.8 ± 0.6, 18.4 ± 1.3 and 18.0 ± 2.7 nmol/10^6^ cells, respectively (Fig. 3d). When 293/mock and 293/B5-11 cells were treated with 100 μM BSO for 2 days, the peaks at 20 min disappeared (Fig. 3e and f, respectively). This excludes the possibility of contamination of compounds other than BSO in the 20 min peak of 293/B5-11 and 293/mb5-8 cells. Therefore, these results clearly showed the upregulation of cellular GSH in *ABCB5*- and *Abcb5*-transfected cells.

The GSH transport by ABCB5 was evaluated using cellular and vesicular transport assays. We first examined the cellular uptake of [^3^H]GSH by 293/mock and 293/B5-11 cells; however, no significant uptake was observed (Fig. 3g). In the vesicle transport assay shown in Fig. 3h, an ATP-dependent uptake of GSH was observed; however, no significant difference was detected between 293/mock and 293/B5-11 vesicles. This suggests that GSH is not a substrate of ABCB5.

**Fig. 4:**
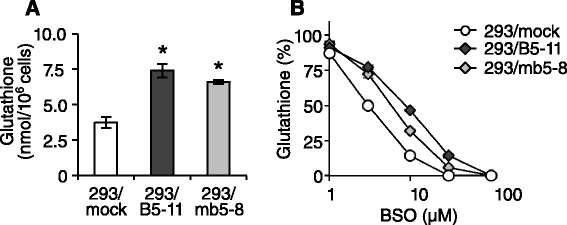
Effect of BSO on cellular glutathione content in *ABCB5*- and *Abcb5*-transfected cells. Cellular glutathione content was determined using a glutathione assay kit. Cells were cultured for 2 days in the absence or presence of various concentrations of BSO. **a** Cellular glutathione content in 293/mock, 293/B5-11 and 293/mb5-8 cells in the absence of BSO. **b** Cellular glutathione content in 293/mock, 293/B5-11 and 293/mb5-8 cells incubated with various concentrations of BSO for 2 days. Data are presented as relative to the value in the absence of BSO. The data are presented as mean ± SD of triplicate determinations. Asterisks show *p* < 0.05 by two-sided *t*-test

We then examined whether the BSO resistance of 293/B5-11 and 293/mb5-8 cells is attributed to the effect of BSO on the depletion of cellular glutathione. 293/mock and 293/B5-11 cells were cultured for 2 days in the absence or presence of various concentrations of BSO, and the cellular glutathione content was measured using an enzyme-based assay kit. In this experiment, the cellular glutathione content in the absence of BSO in 293/mock, 293/B5-11 and 293/mb5-8 cells was 3.7 ± 0.4, 7.4 ± 0.5 and 6.6 ± 0.1 nmol/10^6^ cells, respectively (Fig. 4a). This result confirmed the upregulation of cellular glutathione content in *ABCB5*- and *Abcb5*-transfected cells. As shown in Fig. 4b, the cellular glutathione content was lower in 293/mock cells than in 293/B5-11 cells in each BSO concentration. The BSO concentration required for reducing the glutathione content by 50 % in 293/mock, 293/B5-11 and 293/mb5-8 cells was 3.0 ± 0.2 μM, 8.7 ± 0.2 μM and 5.8 ± 0.3 μM, respectively, indicating that 293/B5-11 and 293/mb5-8 cells were 2.9- and 2.0-fold more resistant to the glutathione-depleting effect of BSO than 293/mock cells, respectively. These results suggest that the BSO resistance of 293/B5-11 and 293/mb5-8 cells can be attributed to the lower effect of BSO on the depletion of cellular glutathione content.

### Glutathione metabolism and glutathione precursor amino acids

**Fig. 5:**
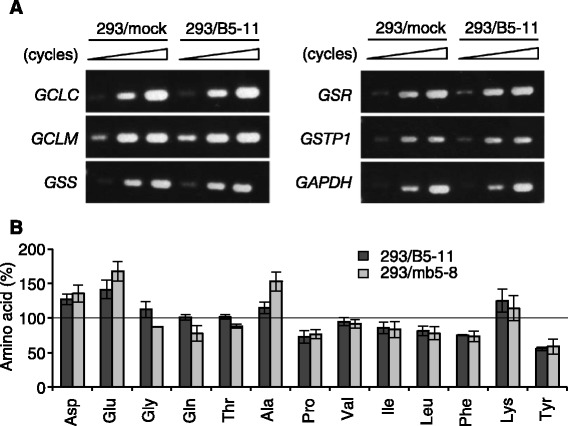
Glutathione metabolism of *ABCB5*- and *Abcb5*-transfected cells. **a** mRNA expression of genes encoding enzymes involved in glutathione metabolism. PCR cycles were as follows: *GCLC*, 29, 32 and 35; *GCLM*, 27, 30 and 33; *GSS*, 29, 32 and 35; *GSR*, 27, 30 and 33; *GSTP1*, 27, 30 and 33; *GAPDH*, 20, 22 and 24. **b** Cellular amino acid contents in 293/B5-11 and 293/mb5-8 cells. The methanol extracts of cells were derivatized using 4-fluoro-7-nitro-2,1,3-benzoxadiazole and subjected to HPLC. Data represent the percentage relative to 293/mock cells for each amino acid. The data are presented as mean ± SD of triplicate determinations

A possible reason for the high GSH content in the *ABCB5*- and *Abcb5*-transfected cells is alteration in the expression of genes encoding glutathione-metabolizing enzymes. To examine this, mRNA expression was evaluated using SurePrint G3 Human GE 8 × 60K Microarray; however, we could not find any genes involved in glutathione synthesis and metabolism whose mRNA expression was significantly altered in 293/B5-11 cells (data not shown). RT-PCR experiments confirmed that there was no significant difference in the expression of *GCLC*, *GCLM*, *GSS*, *GSR* and *GSTP1* mRNA between 293/mock and 293/B5-11 cells (Fig. 5a). GCLC and GCLM are catalytic and modifier subunits of GCL.

GSH is synthesized from Glu, Cys and Gly [17]. We therefore measured the cellular amino acid contents in the *ABCB5*- and *Abcb5*-transfected cells. Figure 5b demonstrates the relative amounts of 13 amino acids that could be quantified by HPLC analysis. The amounts of these amino acids in 293/mock, 293/B5-11 and 293/mb5-8 cells are listed in Table 2. Among the GSH component amino acids, the cellular content of Glu was upregulated in 293/B5-11 and 293/mb5-8 cells (Fig. 5b). The amount of Gly in 293/B5-11 and 293/mb5-8 cells was similar to that in 293/mock cells. The amount of Cys could not be quantified. We also tried to measure the Cys content using other derivatizing reagents, such as AQC and 4-fluoro-7-sulfobenzofurazan; however, a Cys peak was not detected. These results suggest that the increase in the cellular content of Glu was one of the reasons for the upregulation of GSH content in the *ABCB5*- and *Abcb5*-transfected cells. In this experiment, we also observed upregulation of Asp and Ala, and downregulation of Pro, Phe and Tyr in 293/B5-11 and 293/mb5-8 cells (Fig. 5b). These results also suggest the possible effect of ABCB5/Abcb5 on the cellular contents of specific amino acids.

**Table 2:** Cellular amino acids’ content in 293/mock, 293/B5-11 and 293/mb5-8 cells

Amino acid	293/mock	293/B5-11	293/mb5-8
Asp	1.9 ± 0.45 (100)	2.4 ± 0.42 (130)	2.6 ± 0.40 (130)
Glu	8.3 ± 1.5 (100)	12 ± 1.3 (140)	14 ± 1.2 (170)
Gly	4.3 ± 0.28 (100)	4.9 ± 0.82 (110)	3.8 ± 0.24 (87)
Gln	54 ± 7.5 (100)	55 ± 7.5 (100)	42 ± 4.2 (77)
Thr	5.6 ± 0.86 (100)	5.7 ± 0.75 (100)	4.9 ± 0.59 (88)
Ala	0.91 ± 0.12 (100)	1.0 ± 0.19 (110)	1.4 ± 0.15 (150)
Pro	1.9 ± 0.35 (100)	1.4 ± 0.25 (72)	1.4 ± 0.20 (76)
Val	1.6 ± 0.071 (100)	1.5 ± 0.025 (94)	1.5 ± 0.075 (92)
Ile	1.6 ± 0.087 (100)	1.4 ± 0.13 (86)	1.3 ± 0.18 (83)
Leu	1.6 ± 0.23 (100)	1.3 ± 0.14 (81)	1.2 ± 0.18 (78)
Phe	1.1 ± 0.087 (100)	0.80 ± 0.058 (75)	0.78 ± 0.060 (73)
Lys	0.27 ± 0.019 (100)	0.33 ± 0.031 (120)	0.30 ± 0.063 (110)
Tyr	1.0 ± 0.19 (100)	0.57 ± 0.085 (55)	0.60 ± 0.079 (57)

The methanol extracts of the cells were derivatized using 4-fluoro-7-nitro-2,1,3-benzoxadiazole and subjected to HPLC analysis. Each value represents the amino acid’s content (nmol/10^6^ cells). Numbers in parentheses are the percentage relative to 293/mock cells. The data are presented as mean ± SD of triplicate determinations

## Discussion

Several mRNA isoforms of human *ABCB5* have been reported to date [18–21]. Of these, *ABCB5β* mRNA, which is highly expressed in human melanoma cells, encodes an 812-amino acid polypeptide with an N-terminal hydrophobic region with six predicted transmembrane segments and a C-terminal nucleotide-binding region [18, 21–23]. In a previous study, we have identified another mRNA isoform of *ABCB5* that shares a strong homology with ABCB1 and encodes a full-sized ABC transporter that consists of two homologous halves, each containing a hydrophobic region with six predicted transmembrane segments and a nucleotide-binding region. The full-length human *ABCB5*-transfected 293/B5-11 cells showed resistance to doxorubicin, paclitaxel and docetaxel [9]. Expression of full-length ABCB5 in *Saccharomyces cerevisiae* conferred resistance to rhodamine 123, daunorubicin and FK506 [24]. These results suggest that the full-length ABCB5 functions as a multidrug efflux pump.

In the present study, we further screened potential substrates of ABCB5 and found that *ABCB5*-transfected cells showed higher resistance to BSO, an inhibitor of GCL, than the mock-transfected cells (Fig. 1). The degree of BSO resistance of *ABCB5* transfectants depended on the ABCB5 expression level. Murine *Abcb5*-transfected 293/mb5-8 cells also showed BSO resistance. The *ABCB5*- and *Abcb5*- transfected cells also showed resistance to MSO, another GCL inhibitor that shares strong structural similarity with BSO. These results indicate the direct relationship between ABCB5 expression and BSO resistance. To explore the BSO resistance mechanism of the *ABCB5*- and *Abcb5*-transfected cells, we first examined the possibility that ABCB5/Abcb5 decreases the intracellular accumulation of BSO in the transfectants. However, the BSO uptake levels in 293/B5-11 and 293/mb5-8 cells were similar to those in 293/mock cells (Fig. 2). BSO is a small hydrophilic compound with a molecular mass of 222. We have reported that 293/B5-11 cells showed resistance to docetaxel, doxorubicin, daunorubicin, vincristine, etoposide and actinomycin D [9]. All of these are ABCB1 substrates, which are amphiphilic molecules with a molecular mass of 500–1000. Therefore the result that ABCB5/Abcb5 did not affect the cellular accumulation of BSO may not be controversial to previous findings.

BSO and MSO bind and inhibit GCL. They cause depletion of cellular glutathione, which is the primary mechanism of their cytotoxic effect [25, 26]. We showed that the cellular GSH content in the *ABCB5*- and *Abcb5*-transfected cells was significantly higher than that in the mock-transfected cells (Fig. 3). A transport assay revealed that the upregulation of cellular glutathione was not caused by the ABCB5-mediated transport of GSH. We next examined whether the BSO resistance of 293/B5-11 and 293/mb5-8 cells is attributed to the effect of BSO on the depletion of cellular glutathione. As shown in Fig. 4b, the suppressive effect of BSO on the cellular glutathione content was weaker in the *ABCB5*- and *Abcb5*-transfected cells than in the mock-transfected cells, despite the cells having the same level of intracellular BSO. These results suggest that ABCB5/Abcb5 alters cellular glutathione synthesis or metabolism, and thereby reduces the effect of BSO. ABCC1 is known to export glutathione conjugates such as leukotriene C_4_, and co-export anticancer agents such as doxorubicin with GSH [27, 28]. BSO reduces transport of doxorubicin in ABCC1-overexpressing cells by downregulating the cellular GSH level [28]. The cellular GSH content in *ABCC1*-transfected cells was lower than that in control cells [29]. We found that *ABCC1*-transfected KB/MRP1 cells showed hypersensitivity to BSO compared with the parental KB-3-1 cells (data not shown). These results also suggest that the cellular GSH content correlates with BSO sensitivity. However, *ABCB5*- and *Abcb5*-transfected cells are completely different from *ABCC1*-transfected cells in regard to the effect of BSO. In addition, GSH is a transport substrate of ABCC1, but not of ABCB5/Abcb5.

Glutathione is synthesized from Glu, Cys and Gly by two enzymes, GCL and GSS [17]. GCL is composed of the catalytic subunit, GCLC, and the modifier subunit, GCLM [30]. Co-transfection of *GCLC* and *GCLM* cDNAs into COS cells resulted in high cellular GSH levels [31]. However, our cDNA microarray and RT-PCR analyses showed no differences between 293/mock and 293/B5-11 cells in the mRNA expression of genes encoding glutathione-metabolizing enzymes including *GCLC* and *GCLM* (Fig. 5a). Among the transporters, only the expression of *ABCB5* was different, but again there were no other differences between 293/mock and 293/B5-11 cells (data not shown). Glutathione synthesis is regulated by the availability of its substrates [17]. Therefore, we measured the content of cellular amino acids in the *ABCB5*- and *Abcb5*-transfected cells using HPLC, and found that the Glu content was upregulated in these transfectants (Fig. 5b). This may be one of the reasons for the upregulation of the GSH content in the *ABCB5*- and *Abcb5*-transfected cells. We also observed upregulation of Asp and Ala, and downregulation of Pro, Phe and Tyr in 293/B5-11 and 293/mb5-8 cells (Fig. 5b). These results suggest the possible effect of ABCB5/Abcb5 on the cellular contents of specific amino acids.

Currently, there is accumulating evidence that stem cells or tumor-initiating cells express various transporters. ABCG2 is expressed in hematopoietic stem cells and is a determinant of the side-population phenotype. [7]. ABCG2 exports various toxic substances and xenobiotics out of cells, and therefore protects stem cells from such toxic effects. The CD44 variant isoform expressed in cancer stem cells interacts with xCT, a subunit of cystine/glutamate antiporter, promotes glutathione synthesis and increases the cellular GSH level [32]. GSH plays a major role in cellular defenses against reactive oxygen species, and therefore the expression of the CD44 variant isoform protects the cells from oxidative stress [32–34]. ABCB5 is expressed in limbal stem cells and is required for corneal development and repair [11]. ABCB5 has also been reported to be expressed in human melanoma tumor-initiating cells [10]. ABCB5-positive melanoma cells inoculated into immunodeficient mice showed greater tumorigenic capacity than the ABCB5-negative melanoma cells [10]. These results suggest that ABCB5 protects stem cells and tumor-initiating cells from the toxic effect of various poisons. The identification and analysis of cancer stem cell markers are of great interest in current cancer cell biology and are likely to contribute to the development of new strategies for cancer treatment.

## Conclusions

Human *ABCB5*- and murine *Abcb5*-transfected cells showed resistance to BSO, an inhibitor of glutathione synthesis. BSO was not a transport substrate of ABCB5. The cellular glutathione content in the *ABCB5/Abcb5*-transfected cells was significantly higher than that in the mock-transfected cells, because the *ABCB5/Abcb5*-transfected cells were more resistant to the glutathione-depleting effect of BSO than the mock-transfected cells. The current results suggest that ABCB5/Abcb5 upregulates cellular glutathione levels, and thereby protects cells from various poisons.

## Abbreviations

ABC: ATP-binding cassette
GSH: Reduced glutathione
BSO: Buthionine sulfoximine
GCL: Gamma-glutamylcysteine ligase
GAPDH: Glyceraldehyde 3-phosphate dehydrogenase
AQC: 6-aminoquinolyl-N-hydroxysuccinimidyl carbamate
GSS: Glutathione synthase
MSO: Methionine sulfoximine.
